# An outbreak investigation of congenital rubella syndrome in Solomon Islands, 2013

**DOI:** 10.5365/WPSAR.2015.6.4.005

**Published:** 2016-02-03

**Authors:** Kara N Durski, Carol Tituli, Divi Ogaoga, Jennie Musto, Cynthia Joshua, Alfred Dofai, Jennie Leydon, Eric Nilles

**Affiliations:** a WHO Headquarters, Geneva, Switzerland.; b National Referral Hospital, Honiara, Solomon Islands.; c Ministry of Health and Medical Services, Honiara, Solomon Islands.; d Office of the WHO Representative in Solomon Islands, Honiara, Solomon Islands.; e Victorian Infectious Diseases Reference Laboratory (VIDRL), The Doherty Institute, Melbourne, Victoria, Australia.; f Division of Pacific Technical Support, WHO, Suva, Fiji.

## Abstract

**Introduction:**

During May 2012, a rubella outbreak was declared in Solomon Islands. A suspected case of congenital rubella syndrome (CRS) was reported from one hospital 11 months later in 2013. This report describes the subsequent CRS investigation, findings and measures implemented.

**Methods:**

Prospective CRS surveillance was conducted at the newborn nursery, paediatric and post-natal wards, and the paediatric cardiology and ophthalmology clinics of the study hospital from April to July 2013. Retrospective case finding by reviewing medical records was also undertaken to identify additional cases born between January and March 2013 for the same wards and clinics. Cases were identified using established World Health Organization case definitions for CRS.

**Results:**

A total of 13 CRS cases were identified, including two laboratory-confirmed, four clinically confirmed and seven suspected cases. Five CRS cases were retrospectively identified, including four suspected and one clinically confirmed case. There was no geospatial clustering of residences. The mothers of the cases were aged between 20 and 36 years. Three of the six mothers available for interview recalled an acute illness with rash during the first trimester of pregnancy.

**Discussion:**

Additional CRS cases not captured in this investigation are likely. Caring for CRS cases is a challenge in resource-poor settings. Rubella vaccination is safe and effective and can prevent the serious consequences of CRS. Well planned and funded vaccination activities can prevent future CRS cases.

## Introduction

Infection with rubella virus often causes mild disease characterized by fever and rash. Up to 50% of infections are asymptomatic. (*1*) Serious complications including fetal death and congenital rubella syndrome (CRS) may occur when women are infected early in pregnancy. CRS is characterized by congenital heart disease, deafness, glaucoma, cataracts, mental retardation and other disabilities. CRS may be observed in up to 90% of infants born to mothers infected during the first 10 weeks of gestation. (*2*)

CRS is a burden on countries with limited resources, particularly countries with low rubella vaccination coverage rates. In 2010, the reported rubella incidence in the Western Pacific Region was 26 per million population. (*3*) Available data from 2008 to 2010 indicate that more than 30% of female rubella infections were in the childbearing years from 15 to 44 years of age. (*3*) However, information on the burden of CRS in the Western Pacific Region and globally is scant.

**Fig. 1:**
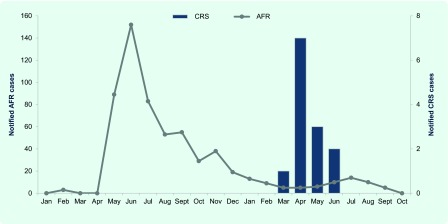
AFR and suspected or confirmed CRS cases by month, Solomon Islands, 2012–2013

AFR, acute fever and rash; and CRS, congenital rubella syndrome.

Solomon Islands (population 515 870 in 2009) is an archipelago consisting of nine provinces and 992 islands located in the Western Pacific. (*4*) Eight provinces have access to a public hospital; in addition there are four private hospitals. In May 2012, a rubella outbreak was declared in Solomon Islands. Six of 10 suspected cases presenting with acute fever and rash (AFR) to a hospital located in the capital city, Honiara (population 64 609 in 2009), (*4*) were laboratory confirmed by rubella-specific immunoglobulin M (IgM) assay. Between May and September 2012, more than 440 cases of AFR were reported through the national syndromic surveillance system, a sentinel surveillance system with eight reporting sites in five provinces at that time (**Fig. 1**). During April 2013, 11 months after the start of the rubella outbreak, a newborn infant with cataracts and thrombocytopenia was reported as a suspected case of CRS by a paediatrician at the hospital. This report describes the subsequent CRS investigation, findings and control measures implemented at the hospital.

## Methods

Prospective CRS surveillance was conducted at the study hospital in the newborn nursery, paediatric and postnatal wards and the paediatric cardiology and ophthalmology clinics from April to July 2013 using established World Health Organization (WHO) guidelines and case definitions. (*5*) We also conducted retrospective case finding from January to March 2013 by reviewing medical records for clinically compatible illnesses and demographics for the same wards and clinics using the same case definitions. The investigation period was based on the estimated gestational period of pregnant women who may have been infected during the 2012 rubella outbreak (1 May to 30 September 2012) as no routine rubella vaccination or CRS surveillance existed in Solomon Islands before this 2012 outbreak.

The following case definitions were used to identify and classify CRS cases. A suspected case of CRS was any infant less than one year of age in whom a health worker suspects CRS, including any infant with heart disease and/or suspicion of deafness and/or one or more of the following eye signs: cataract, diminished vision, nystagmus, squint, microphthalmus or congenital glaucoma. A clinically confirmed case of CRS was any infant less than one year with two complications in group A or one from A and one from B.

- Group A: cataract(s), congenital glaucoma, congenital heart disease, loss of hearing, pigmentary retinopathy; and
- Group B: purpura, splenomegaly, microcephaly, mental retardation, meningoencephalitis, radiolucent bone disease, jaundice with onset within 24 hours after birth.

A laboratory-confirmed case of CRS was a clinically confirmed CRS case with presence of serum anti-rubella IgM (Beckman Access, Lane Cove, Australia) or rubella-specific ribonucleic acid from pharyngeal swabs tested by reverse-transcriptase polymerase chain reaction (RT–PCR). Mothers were asked if they had illnesses with rash during their pregnancies. Infants had serum and pharyngeal swabs collected for testing. Serum was not available for the retrospectively identified cases and laboratory testing was not performed.

This investigation obtained WHO ethics approval (2015.16.SOL.2.ESR).

## Results

In total 13 CRS cases were identified during the investigation period. All CRS cases were born within a gestational period from the rubella outbreak in 2012 (**Fig. 1**). Eight CRS cases were prospectively identified including three suspected, three clinically confirmed and two laboratory-confirmed cases; six cases were identified in the nursery and two cases, who presented with cataracts, in the outpatient paediatric clinics. The two laboratory-confirmed CRS cases were anti-rubella IgM-positive (of which one was also RT–PCR-positive); the remaining six cases were anti-rubella IgM-negative (**Table 1**). The mothers were aged between 20 and 36 years. There was no geospatial clustering of residences. Three of the six (50%) mothers interviewed recalled an acute illness with rash during the first trimester of pregnancy; no other serious illness was reported during pregnancy.

**Table 1:** Prospectively and retrospectively identified cases of suspected, clinically confirmed and laboratory-confirmed CRS patients at one hospital in Honiara, Solomon Islands, 2013

Prospectively/ retrospectively identified	Sex	Age of case when examined	Birth weight (kg)	Clinical features	Anti-rubella IgM	Outcome	Birth year of mother	Fever and/or rash during first trimester	Classification
Prospective	Male	Day 0	1.50 FT	CC, TCP	Positive	Discharged	1993	Yes	Laboratory-confirmed
Prospective	Female	Day 0	2.73	TCP, PR	Negative	Discharged	1987	No	Suspected
Prospective	Female	Day 0	3.17 FT	CC, TCP, ENC	Negative	Death	1988	Yes	Clinically confirmed
Prospective	Male	Day 0	1.97	CC	Positive^†^	Discharged	1994	Yes	Laboratory-confirmed
Prospective	Male	Day 0	3.00 FT	CC, CHD	Negative	Discharged	1992	No	Clinically confirmed
Prospective	Female	Day 0	3.05 FT	CC, CHD	Sample not tested	Discharged	1977	Yes	Clinically confirmed
Prospective	Male	6 months*	N/A	CC	Negative	N/A	N/A	N/A	Suspected
Prospective	Male	6 months*	N/A	CC	Negative	N/A	N/A	N/A	Suspected
Retrospective	Male	Day 0	1.50 FT	TCP, IUGR	Not tested	Death	N/A	N/A	Suspected
Retrospective	Female	Day 0	N/A	N/A	Not tested	Death	N/A	N/A	Suspected
Retrospective	N/A	Day 0	N/A	TCP, PR	Not tested	Discharged	N/A	N/A	Suspected
Retrospective	N/A	Day 0	N/A	CC	Not tested	Discharged	N/A	N/A	Suspected
Retrospective	N/A	Day 0	N/A	CC, CHD	Not tested	Discharged	N/A	N/A	Clinically confirmed

CC, congenital cataracts; CHD, congenital heart disease [clinical diagnosis by the hospital paediatricians]; ENC, encephalitis; FT, full term; IGUR, intrauterine growth retardation; N/A, not available; PR, petechial rash; and TCP, thrombocytopenia.

* Sample collected at 6 months of age.

^†^ Both IgM and RT–PCR positive.

Five CRS cases were retrospectively identified by medical record review and/or paediatricians’ recall, including four suspected and one clinically confirmed case. The first suspected CRS case was born on 5 March 2013 and diagnosed with intrauterine growth retardation, overwhelming sepsis, thrombocytopenia, severe anaemia and asphyxia. From 5 March to 9 April, four newborns were admitted to the nursery with clinical characteristics of CRS, including purpuric rash, cataracts and/or congenital heart disease. Two of the five infants died shortly after birth (**Table 1**).

## Discussion

This is the first documented CRS outbreak in Solomon Islands. In 2012, the Solomon Islands Ministry of Health and Medical Services (MHMS) implemented indicator- and event-based early warning outbreak disease surveillance as part of the Pacific Syndromic Surveillance System that includes weekly reporting and investigation of AFR cases. (*6*) There were eight sentinel sites in five of nine provinces in 2012. From May to September 2012, unusual and substantial increases in AFR were documented from all sentinel sites. Given the absence of routine rubella vaccination, and given that six out of 10 (60%) samples tested from Honiara were confirmed for rubella, it is probable that widespread rubella transmission occurred during this period. Prior to implementation of the early warning surveillance system in 2012, the rubella outbreak would have likely gone unreported.

Despite the small number of laboratory-confirmed CRS cases, the timing of the CRS outbreak is consistent with the previous rubella outbreak in 2012 (**Fig. 1**). Suspected newborn CRS cases that test anti-rubella IgM negative should be re-tested one month later, as approximately 20% of infected infants may not have detectable titres before one month of age; (*7*) this diagnostic follow-up was not possible as the cases had returned to their villages. Given that substantial rubella transmission appears to have started in May 2012 and prospective CRS surveillance was only implemented from April 2013, it is probable that additional cases went undetected. Additional undetected cases in the other provinces where CRS surveillance was not conducted is also possible.

During 2013, a seroprevalence survey of 100 pregnant women attending the prenatal clinic at the same study hospital was conducted by the MHMS to assess for pre-existing immunity to rubella; 97% of the samples were positive for anti-rubella IgG (MHMS, unpublished data, 2013), demonstrating high rates of prior exposure and infection in this cohort. Given that rubella vaccination was not routinely administered in Solomon Islands until 2013, the high anti-rubella IgG positive proportion suggested substantial prior rubella virus transmission in Honiara. It is not possible to determine if these cases were infected during the 2012 rubella outbreak or during earlier, undocumented rubella transmissions.

CRS is a frequent complication of rubella infection in early pregnancy. (*8*) Preventing future rubella outbreaks and CRS cases in a resource-limited setting requires careful consideration and planning. The immunization coverage in the population should be greater than 80%, with at least one dose of vaccine, to prevent CRS outbreaks. (*5*) A vaccination strategy that achieves partial coverage may decrease but not eliminate rubella transmission, potentially shifting the average age of infection from childhood to adolescence and adulthood and increasing the risk of infection during the child-bearing years. (*5*)

Infants born with CRS are potentially infectious for up to one year. (*9*) In a setting where the susceptible population is unknown and vaccination coverage is low, implementing control measures to avoid the spread of disease is challenging. Important recommendations were implemented to minimize transmission of rubella within the hospital, including reinforcing hand-washing protocols, procuring and stocking hand sanitizing supplies within the nursery, temporarily relocating pregnant staff to other wards and isolating infectious cases. (*10*) Prior to hospital discharge, health-care workers must educate families about how to prevent transmission of rubella to others, in particular avoiding contact between pregnant women and the infectious infant.

Caring for CRS cases is a challenge in resource-poor settings. A CRS outbreak has a long-standing impact on vulnerable populations with minimal access to cardiac, auditory and ophthalmologic services. Rubella vaccination is safe and effective. (*5*) Well planned and funded vaccination activities can prevent future CRS cases, including in resource-poor countries.
